# RNA amplification for successful gene profiling analysis

**DOI:** 10.1186/1479-5876-3-28

**Published:** 2005-07-25

**Authors:** Ena Wang

**Affiliations:** 1 Immunogenetics Section, Department of Transfusion Medicine, Clinical Center, National Institutes of Health, Bethesda, MD, 20892, USA

**Keywords:** Gene profiling, cDNA microarray, RNA amplification, polymerase, high throughput analysis.

## Abstract

The study of clinical samples is often limited by the amount of material available to study. While proteins cannot be multiplied in their natural form, DNA and RNA can be amplified from small specimens and used for high-throughput analyses. Therefore, genetic studies offer the best opportunity to screen for novel insights of human pathology when little material is available. Precise estimates of DNA copy numbers in a given specimen are necessary. However, most studies investigate static variables such as the genetic background of patients or mutations within pathological specimens without a need to assess proportionality of expression among different genes throughout the genome. Comparative genomic hybridization of DNA samples represents a crude exception to this rule since genomic amplification or deletion is compared among different specimens directly. For gene expression analysis, however, it is critical to accurately estimate the proportional expression of distinct RNA transcripts since such proportions directly govern cell function by modulating protein expression. Furthermore, comparative estimates of relative RNA expression at different time points portray the response of cells to environmental stimuli, indirectly informing about broader biological events affecting a particular tissue in physiological or pathological conditions. This cognitive reaction of cells is similar to the detection of electroencephalographic patterns which inform about the status of the brain in response to external stimuli. As our need to understand human pathophysiology at the global level increases, the development and refinement of technologies for high fidelity messenger RNA amplification have become the focus of increasing interest during the past decade. The need to increase the abundance of RNA has been met not only for gene specific amplification, but, most importantly for global transcriptome wide, unbiased amplification. Now gene-specific, unbiased transcriptome wide amplification accurately maintains proportionality among all RNA species within a given specimen. This allows the utilization of clinical material obtained with minimally invasive methods such as fine needle aspirates (FNA) or cytological washings for high throughput functional genomics studies. This review provides a comprehensive and updated discussion of the literature in the subject and critically discusses the main approaches, the pitfalls and provides practical suggestions for successful unbiased amplification of the whole transcriptome in clinical samples.

## Introduction

Quantification of gene expression is a powerful tool for the global understanding of the biology underlying complex pathophysiological conditions. Advances in gene profiling analysis using cDNA or oligo-based microarray systems uncovered genes critically important in disease development, progression, and response to treatment [1-12]. While the expression of a single or a limited number of genes can be readily estimated using minimum amount of total or messenger RNA (mRNA) from experimental or clinic samples, gene profiling requires large amount of RNA which can only be generated from global RNA amplification when using often limited amount clinical material. Conventionally at least 50 – 100 μg of total RNA (T-RNA) or 2 – 5 μg poly(A)^+ ^RNA are generally necessary for global transcript analysis studies though efforts to enhance signal intensity and fluorochrome incorporation have reduced the amount of total RNA needed for array analysis to 1–5 ug [13]. Large amounts of RNA are not usually obtainable from clinical specimens. Thus, they pertain to experimental endeavors where cultured cell lines or tissues from pooled experimental models are used while only occasionally they are obtainable from large excisional biopsies [14]. However, most biological specimens directly obtained *ex vivo *for diagnostic or prognostic purposes or for clinical monitoring of treatment are too scarce to yield enough RNA for high throughput gene expression analysis. Needle or punch biopsies provide the opportunity to serially sample lesions during treatment or to sample lesion to identify predictors of treatment outcome by observing the fate of the lesion left in place. In addition, the simplicity of the storage procedure associated with the collection of small samples which can be performed at the bed side provides superior quality of RNA with minimum degradation [15]. Finally, the hypoxia which follows ligation of tumor-feeding vessels before excision is avoided with these minimally invasive methods, therefore, obtaining a true snapshot of the *in vivo *transcriptional program. These minimally invasive sampling techniques yield generally few micrograms of total RNA and most often even less [15,16]. Similarly, breast and nasal lavages and cervical brush biopsies, routinely used for pathological diagnosis, generate insufficient material far below the detection limit of most assays. Acquisition of cell subsets by fluorescent or magnetic sorting or laser capture micro-dissection (LCM) for a more accurate portraying of individual cell interactions in a pathological process generate even less material, in most cases, nanograms of total RNA [17-20].

Efforts have been made to broaden the utilization of cDNA microarrays using two main strategies: intensifying fluorescence signal [13,21-24] or amplifying RNA. Signal intensification approaches have reduced the requirement of RNA few folds but cannot extend the utilization of microarray to sub-microgram levels. RNA amplification in turn has gained extreme popularity based on amplification efficiency, linearity and reproducibility lowering the amount of total RNA needed for microarray analysis to nanograms without introducing significant biases. Methods aimed at the amplification of poly(A)-RNA [25] via *in vitro *transcription (IVT) [26] or cDNA amplification via polymerase chain reaction (PCR) [27] have reduced the material needed for cDNA microarray application and extended the spectrum of clinical samples that can be studied. Nanograms of total RNA have been successfully amplified into micrograms of pure mRNA for the screening of the entire transcriptome without losing the proportionality of gene expression displayed by the source material. Curiously, the most important advances were made by Eberwine whose main goal was not to use clinical material for high-throughput studies but rather to amplify enough material from single cells for individual or few gene analysis [28,29]. His revolutionary contribution has, however, provided a striking opportunity to explore the function of the human genome *ex vivo *and has exponentially opened the frontiers of clinical investigation. Modifications, optimizations and validations of RNA amplification technology based on Eberwine's pioneering work are still actively explored.

In this chapter, we will summarize efforts to optimize RNA amplification and describe in detail current amplification procedures that have been validated and applied to cDNA microarray analysis.

### Collection of source material and RNA isolation

Samples used for RNA isolation and amplification should always be collected fresh and immediately processed. Excisional biopsies should be handled within 20 min and stored at -80°C (for instance with RNAlater™, Ambion, Austin, TX) if RNA isolation cannot be performed right away. Material from FNA should be collected in 5 ml of ice cold 1 × PBS or other collection medium without serum at the patient's bedside to minimize RNA metabolism or degradation. After spinning at 1,500 rpm for 5 minutes at 4°C, 2.5 ml of ACK lysing buffer should be added with 2.5 ml of 1 × PBS and incubated for 5 minutes on ice to lyse red blood cells (RBC) in case of excessive contamination. Cell pellets should be washed in 10 ml 1 × PBS and then re-suspend in small volumes of RNAlater followed by snap freezing or prior lysis of the pellet in 350 μl of RLT buffer with fresh addition of 2-mercaptoethanol (2-ME) (RNeasy mini kit, QIAGEN Inc, Valencia, CA USA) before snap freezing at -80°C. For LCM, good results can be obtained by lysing cells directly in 50 μl RLT buffer with 2-ME. Total RNA (T-RNA) and poly A RNA can both be used as starting material for RNA amplification.

The RNA isolation method strongly affects the quality and quantity of RNA. T-RNA can be isolated using commercially available RNA isolation kits. The T-RNA content per mammalian cell ranges between 20 to 40 pg of which only 0.5 – 1.0 pg are constituted by messenger RNA (mRNA) [30,31]. Sample condition, viability, functional status and phenotype of the cells are the major reasons for differential yield of T-RNA. Sample handling with precaution for RNase contamination always improves the quality and quantity of the RNA obtained. Measurement of T-RNA concentration can be performed with a spectrophotometer at OD_260_. An OD_260/280 _ratio above 1.8 is to be expected. When a very limited number of cells is available such as from LCM or FNA, very low or even negative OD readings may be observed. In this case, OD reading can be omitted. When RNA is isolated from archived samples or from samples whose collection and storage conditions were not controlled and optimized, it is preferable to estimate RNA quality and quantity using Agilent Bioanalyzer (Agilent Technologies Inc. Palo Alto, CA) or RNA gels. Clear 28S and 18S ribosomal RNA bands indicate good quality of RNA. Since 28S rRNA degradation occurs earlier than 18S rRNA and mRNA degradation in most cases correlates with 28S ribosomal RNA, the ratio of 28S versus 18S rRNA is a good indicator of mRNA quality [32]. 28S/18S rRNA ratios equal or close to 2 suggest good RNA quality.

### Single strand cDNA synthesis

A critical step in RNA or cDNA amplification is the generation of double stranded cDNA (ds-cDNA) templates. First strand cDNAs are reverse transcribed from mRNA using oligo dT or random primers. In order to generate full length first strand cDNA, oligo dT(15–24 nt) with an attachment of a bacterial phage T7 promoter sequence is commonly used to initiate the cDNA synthesis [25,29,33-36]. In case of degraded RNA [37], random primers with attachment of T3 RNA polymerase promoter (T3N9) have been used for first and second strand cDNA synthesis [38]. To prevent RNA degradation while denaturing and during the reverse transcription (RT) reaction, it is useful to denature the RNA (65°C for 5 minutes or 70°C for 3 minutes) in the presence of RNasin^® ^Plus RNase Inhibitor (Promega, Madison, WI) which forms a stable complex with RNases and inactivates RNase at temperatures up to 70°C for at least 15 minutes.

To enhance the efficiency of the RT reaction and reduce incorporation errors, the temperature of the RT reaction can be maintained at 50°C [39,40] instead of 42°C to avoid the formation of secondary mRNA structures. This can be done by using thermo-stable reverse transcriptase (ThermoScript™ RNase H- Reverse Transcriptase, Invitrogen, Carlsbad, CA) or regular RTase [41] in the presence of disaccharide trehalose [42-44]. Disaccharide trehalose not only can enhance the thermo-stability of RTase but also posses thermo-activation functions. This modification greatly enhances the accuracy and the efficiency of RT with minimum impact on the DNA polymerase activity [39]. The utilization of DNA binding protein T4gp32 (USB, Cleveland) in RT reactions also improves cDNA synthesis [40,41,45,46]. T4gp32 protein may essentially contribute to the qualitative and quantitative efficiency of the RT reaction by reducing higher order structures of RNA molecules and hence reduce the pause sites during cDNA synthesis.

In Van Gelder and Eberwine's T7 based RNA amplification [28], the amount of oligo dT-T7 primer used in the first strand cDNA synthesis can affect the amplified RNA in quantity and quality. Excessive oligo dT-T7 in the RT reaction could lead to template independent amplification [47]. This phenomenon is not observed when the template switch approach is combined to *in vitro *transcription (Wang, E. unpublished data).

### Double stranded cDNA (ds-cDNA) synthesis

RNA amplification methods differ according to the strategies used for the generation of ds-cDNA as templates for *in vitro *transcription or PCR amplification. There are two basic strategies that have been extensively validated and applied for high throughput transcriptional analysis. The first is based on Gubler-Hoffman's [48] ds-cDNA synthesis subsequently optimized by Van Gelder and Eberwine [28,29]. This technology utilizes RNase H digestion to create short fragments of RNA as primers to initiate the second strand cDNA elongation under DNA polymerase I. Fragments of second strand cDNA are then ligated to each other sequentially under *E. Coli *DNA ligase followed by polishment using T4 DNA polymerase to eliminate loops and to form blunt ends. Amplifications based on this methods have been widely used in samples obtained in physiological or pathological conditions and extensively validated for its fidelity, reproducibility and linearity compared to un-amplified RNA from the same source materials [29,33,47,49-52].

The alternative ds-cDNA synthesis approach utilizes retroviral RNA recombination as a mechanism for template switch to generate full length ds-cDNA. The method was initially invented for full length cDNA cloning and, therefore, the main targets of this method are undegredated transcripts. Gubler-Hoffman's ds-cDNA synthesis has the potential of introducing amplification biases because of a possible 5' under-representation. In addition, the low stringency of the temperature in which ds-cDNA synthesis occurs may introduce additional biases [33]. Although 5' under-representation could, in theory, be overcome by hairpin loop second-strand synthesis [53], the multiple enzymes (4) used in the reaction could also in turn cause errors.

To ensure generation of full-length ds-cDNA, [54] synthesis is performed taking advantage of the intrinsic terminal transferase activity and template switch ability of Moloney Murine Leukemia Virus RTase [55]. This enzyme adds non-template nucleotides at the 3' end of the first strand cDNA, preferentially dCTP oligo nucleotides. A template-switch oligonucleotide (TS primer) containing a short string of dG residues at the 3' end is added to the reaction to anneal to the dC string of the newly synthesized cDNA. This produces an overhang that allows the RTase to switch template and extend the cDNA beyond the dC to create a short segment of ds-cDNA duplex. After treatment with RNase H to remove the original mRNA, the TS primer initiates the second stranded cDNA synthesis by PCR. Since the terminal transferase activity of the RTase is triggered only when the cDNA synthesis is complete, only full-length single stranded cDNA will be tailed with the TS primer and converted into ds-cDNA. Using the TS primer, second strand cDNA synthesis is carried at 75°C after a 95°C denaturing and a 65°C annealing step in the presence of single DNA polymerase [35]. This technique, in theory, overcomes the bias generated by amplification methods depending only on 3' nucleotide synthesis and hence it is, in theory, superior to the Gubler-Hoffman's ds-cDNA synthesis. However, no significant differences in correlation coefficients of amplified versus non amplified RNA were observed when the Gubler-Hoffman's ds-cDNA method was compared with the TS ds-cDNA amplification using high throughput analysis [40,56] The fidelity of template switch-based amplification methods has been assessed by numerous gene profiling analyses on different type of microarray platforms, real time PCR and sophisticated statistical analyses and it has been well accepted for high throughput transcriptome studies.

### RNA amplifications

#### Linear amplification

Amplification of mRNA without skewing relative transcript abundance remains a focus of research. Linear amplification methods have been developed that in theory should maintain the proportionality of each RNA species present in the original sample. IVT using ds-cDNA equipped with a bacteriophage T7 promoter [28] provides an efficient way to amplify mRNA sequences and thereby generate templates for synthesis of fluorescently-labeled single-stranded cDNA [25,26,28,29,33,53]. Depending upon the T7 or other (T3 or SP6) promoter sequence position on the ds-cDNA, amplified RNA can be either in sense or antisense orientation. Oligo dT attachments to the promoter sequence, for example oligo dT-T7, prime first strand cDNA positioned the promoter at the 3' end of genes (5' end of cDNA) and, therefore, lead to the amplification of antisense RNA (aRNA) or complement RNA (cRNA). Promoters positioned at the 5' end of genes by random [57] or TS primers (Wang E, unpublished observation) generate sense RNA (sRNA). Amplified sRNA can be also produced by tailing of oligo dT to the 3' of the cDNA followed by oligo dA-T7 priming for double stranded T7 promoter generation at the 5' end of genes [58]. The singularity of this approach resides in the utilization of a DNA polymerase blocker at the 3' of the oligo dA-T7 primer which prevents the elongation of second strand cDNA synthesis while priming for the elongation of the double stranded promoter. In this fashion, only sense amplification can be achieved by the presence of the 5' ds-T7 promoter followed by single strand cDNA templates.

IVT using DNA-dependent RNA polymerase is an isothermal reaction with linear kinetics. The input ds-cDNA templates are the only source of template for the complete amplification and, therefore, any errors created on the newly synthesized RNA will not be carried or amplified in the following reactions. Overall, RNA polymerase makes an error at a frequency of about once in 10,000 nucleotides corresponding to about once per RNA strand created . This contrasts with DNA-dependent DNA polymerase which incorporates an error once in every 400 nucleotides. Most importantly, these errors are exponentially amplified in the following reaction since the amplicons serve as templates. Thus, RNA polymerase catalyzes transcription robotically and efficiently without sequence dependent bias. Recombinant RNA polymerases have been engineered to enhance the stability of the enzyme interacting with templates and reduce the abortive tendency [59] of the wild type RNA polymerase which in turn improved the elongation phase resulting in complete mRNA transcripts. The length of amplified RNA ranges from 200 to 6,000 nucleotides for the first round of amplification and 100 to 3,000 nucleotides for the second round when random primers are used [36,60] The amplification efficiency is greater than 2,000 fold in the first round and 100,000 fold in the second round [35,60]

Two rounds of IVT are commonly required when sub micrograms of input total RNA are used. It has been estimated that after two rounds of amplification the frequency of only 10% of the genes in a specimen is reduced [61] and more than two rounds of amplification may still retain at least in part the proportionality of gene expression among different RNA populations [35]. However, we, generally, do not recommend going over two rounds of amplification unless necessary for extremely scant specimens such as when processing single or few cell specimens, to avoid unnecessary biases related to amplification. The fidelity of IVT has been extensively assessed by gene profiling analysis, quantitative real-time PCR and statistical testing by comparing estimates of gene expression in amplified versus non-amplified RNA [35].

Pitfalls have been also associated with IVT. The fidelity of the first round amplification decreases when the input starting material is less than 100 ng because of the intrinsic low abundance of transcripts (particularly those under represented in the biological specimen). This can be rescued by two rounds of IVT if sufficient RNA species are present in the input material [35]. In addition, two rounds of amplification tend to introduce a 3' bias due to the use of random primers in the cDNA synthesis for ds-cDNA template creation. This should not affect the usefulness of the technique for high throughput gene profiling analysis since cloned cDNA arrays are 3' biased and even oligo arrays are designed to target the 3' end of each gene. Sequence-specific biases introduced during amplification are generally reproducible and, although negligible, could mislead data interpretation only when amplified RNA is directly compared with non amplified RNA on the same array platform. This type of error can be easily circumvented by using samples processed in identical conditions. Degradation of amplified RNA during prolonged (more than 5 hours) IVT may result in lower average size of aRNA and decreased yields [37]. This results from residual RNase in the enzyme mixture used for IVT reaction and can be prevented by the addition of RNase inhibitor in the reaction if a prolonged amplification is needed.

#### PCR-based exponential amplification

IVT is burdensome time consuming and may, theoretically, produce a 3' bias especially when two rounds of amplification are employed. Exponential amplification (PCR-based) may avoid these drawback and it has shown promise since, contrary to the IVT, is simple and efficient. However, PCR-based amplification has its own drawbacks.

The limitations of PCR-based amplification stem from the characteristics of the DNA-dependent DNA polymerase enzymatic function. The function of this enzyme is biased towards a lower efficiency in the amplification of GC rich sequences compared with AT rich sequences. In addition, as previously discussed, not only creates errors more frequently than RNA polymerase but also amplifies these mistakes because the reaction utilizes the amplicons as templates for subsequent amplification [62]. In addition, due to the exponential amplification, the reaction could reach saturation in conditions in which excess input template quantities are used or because of the exhaustion of substrate. This would favor the amplification of high-abundance transcripts which would compete more efficiently for substrate in the earlier cycles of the amplification process resulting in loss of proportionality of the amplification process. Optimization of PCR cycle number to avoid reaching the saturation cycle and adjustments in the amount of template input could overcome the problems [63]. The utilization of DNA polymerase with proofreading function could eradicate errors created in the cDNA amplification [64]. This approach preserves the relative abundance of transcript [65] and it may outperform IVT when less than 50 ng of input RNA are available as starting material [66,67].

PCR-based cDNA amplification can be categorized as template switching (TS)-PCR [52,68,69], random PCR [70] and 3' tailing with 5' adaptor ligation PCR [71] based on the generation of a 5' anchor sequence which provides a platform for 5' primer annealing. TS-PCR employs the same template switch mechanism in ds-cDNA generation and in the amplification of ds-cDNA using 5' TS primer II (truncated TS primer) and 3' oligo dT or dT-T7 primers (depending upon the primer used in the first strand cDNA synthesis). Random PCR utilizes modified oligo dT primers (dT-T7 or dT-TAS (**T**arget **A**mplification **S**equence) or random primers with an adaptor sequence for the first strand cDNA initiation and random primers with an attachment of the same adaptor, for example dN10-TAS [70], for second strand cDNA synthesis. The attached sequence, such as TAS, generates a 5' anchor on the cDNA for subsequent PCR amplification with a single TAS-PCR primer. This approach is more suitable for RNA with partial degradation and with the risk of under representation of the 5' end. The third exponential amplification utilizes terminal deoxynucleotidyl transferase function to add a polymonomer, for example poly dA, tail to the 5' end of the gene. The tailed poly dA provides an annealing position for the oligo dT primer which lead the second strand cDNA synthesis. Ds-cDNA can then be amplified under one oligo dT primer or dT-adaptor primer if an adaptor sequence is attached [66]. Direct adaptor ligation is another alternative way to generate ds-cDNA with a known anchor sequence at the 5' end [71]. In this way, single strand cDNA is generated using oligo dT primers immobilized onto magnetic beads and second strand cDNA is completed by Van Gelder and Eberwine's ds-cDNA generation method. A ds-T7 promoter-linker is then unidirectionally ligated to the blunted ds-cDNA at the 5' end. PCR amplification can then be performed using the 5' promoter primer and the 3' oligo dT or dT-adapter primer, if an adapter is attached. PCR amplified ds-cDNA is suitable for either sense or antisense probe arrays.

The combination of PCR amplification to generate sufficient ds-cDNA template followed by IVT [70,71]. is an attractive strategy to amplify minimal starting material since it takes advantage of the efficiency of the PCR reaction and the linear kinetics of IVT while minimizing the disadvantage discussed above. Validations of PCR-based RNA amplification methods are fewer than those for IVT but have been so far persuasive in spite of the prevalent expectations. Skepticism concerning the reproducibility and linearity are still one of the key factors preventing the extensive application of this approach.

### Target labeling for cDNA microarray using amplified RNA

The generation of high quality cDNA microarray data depends not only on sufficient amount and highly representative amplified target, but also on the target labeling efficacy and reproducibility. Steps involved in the targets preparation such as RNA amplification, target labeling, pre-hybridization, hybridization and slides washing are imperative in enhancing foreground signal to background noise ratios. Linear spectrum of signal intensity that correlates with gene copy numbers without having to compensate detection sensitivity is one of the key factors for high quality cDNA analysis. Therefore, target labeling is a critical step to achieve consistently high signal images.

Typically, fluorescently-labeled cDNA is generated by incorporation of conjugated nucleotide analogs during the reverse transcription process. Depending upon the detection system, labeled markers can be either radioactive, color matrix or florescent. Florescence labeling outperforms the other labeling methods because of the versatile excitation and emission wave length. In addition, it has the advantage of not being hazardous. Among the fluorochrome, Cy3 (N, N8-(dipropyl)-tetramethylindocarbocyanine) and Cy5 (N, N8-(dipropyl)-tetramethylindodicarbocyanine) are most commonly used in cDNA microarray applications due to their distinct emission (510 and 664 respectively). Cy5 labeled dUTP and dCTP are less efficient in incorporation during the labeling reaction compared to Cy3 labeled dUTP or dCTP and they are more sensitive to photo bleach because of their chemical structure. Therefore, labeling bias needs to be accurately analyzed and results should be normalized according to standard normalization procedures.

Target labeling can be divided into two major categories: direct fluorescence incorporation and indirect fluorescence incorporation. The first category utilizes fluorescence-labeled dUTP or dCTP to partially substitute unlabeled dTTP or dCTP in the RT reaction to generate Cydye-labeled cDNA. This label incorporation method is suitable for cDNA clone microarray using amplified aRNA as templates or oligo array using amplified sRNA as template.

A limitation of direct labeling consists in the fact that fluorescent nucleotides are not the normal substrates for polymerases and some may be particularly sensitive to the structural diversity of these artificial oligonucleotides. The fluorescent moieties associated with these nucleotides are often quite bulky and, therefore, the efficiency of incorporation of such nucleotides by polymerase tends to be much lower than that of natural substrates. An alternative is to incorporate, either by synthesis or by enzymatic activity, a nucleotide analog similar to the natural nucleotide in structure featuring a chemically reactive group, such as 5-(3-Aminoallyl)-2'-deoxyuridine 5'-triphosphate (aa-dUTP), to which a fluorescent dye, such as Cydye, may then be attached [72]. The reactive amine of the aa-dUTP can be incorporated by a variety of RNA-dependent and DNA-dependent DNA polymerases. After removing free nucleotides, the aminoallyl labeled samples can coupled to dye, purified again, and then applied to a microarray [73]. The optimized ratio of aa-dUTP versus dTTP in the labeling reaction should be 2 to 3 respectively.

In theory, indirect outperforms direct labeling by reducing of the cost and maximizing signal intensity through increases in incorporation of fluorochrome or through signal amplification using fluorescence-labeled antibody or biotin-streptavdin complexes. However, more steps are involved in the purification of the labeled target prior to hybridization which make this strategies less frequently used.

### RNA amplification protocols

The protocols presented here are routinely used in our laboratory in response to several inquires by interested investigators, The protocol is based on a combination of strategies discussed in the previous section that have been used for RNA amplification that we have applied to optimize TS-IVT following the original Eberwine's RNA amplification protocols.

### Material and reagents

Dilute stock solution to the appropriate working concentration.

dNTP mix solution (dATP, dCTP, dGTP, dTTP, 10 mM each) (Pharmacia Cat# 27-2035-02)

Low T dNTP (5 mM dA, dG and dCTP, 2 mM dTTP)

RNasin Plus (20 units/μl) (Promega Cat# N2611)

Advantage PCR buffer (come with Advantage cDNA polymerase)

Linear Acrylamide (0.1 ug/μl. Ambion; Cat# 9520)

Phenol: Chloroform: Isoamyl alcohol (25:24:1) (Boehringer Mannhem Cat #101001)

Phase Lock gel (heavy) (5 prime to 3 prime, Inc.; Cat# pl-188233)

7.5 M ammonium acetate (Sigma; Cat# A2706)

DEPC treated H_2_O

In Vitro Tanscription Kit (Ambion; T7 Megascript Kit #1334)

Cy-dUTP (1 mM Cy3 or Cy5)

1 M NaOH

500 mM EDTA

1 × TE

1 M Tris pH 7.5

50× Denhardt's blocking solution (Sigma; Cat# 2532)

Poly dA_40–60 _(8 mg/ml) (Pharmacia; Cat# 27-7988-01)

Human Cot I DNA (10 mg/ml) (Invitrogen; Cat# 15279-011)

20 × SSC

10% SDS

T4gp32 protein (8 mg/ul) (USB Cat# 74029Y)

### Enzymes

RNase H^- ^MMLV Reverse Transcriptase (Superscript II) (200 units/μl) (Invitrogen; Cat# 18064-071)

50× Advantage cDNA Polymerase mix (Clontech Cat# 8417-1)

RNase H (2 U/μl. Invitrogen; Cat# 18021-071)

10× T7 RNA polymerase mix (within the Megascript kit)

### Primers

Oligo dT-T7 primer (5' AAA CGA CGG CCA GTG AAT TGT AAT ACG ACT CAC TAT AGG CGC T_(15) _3') (0.125–0.25 μg/μl for the first round amplification depending on the amount of input total RNA and 0.5 μg/μl for the second round amplification) in RNase free water. Synthesized primer should be SDS-PAGE purified to insure the full length. The concentration of primer is varied according to the starting material used. This promoter sequence is much longer than the consensus sequence defined by Dunn and Studier (1983) and can be purchased from New England Biolabs and Stratagene Inc. In the extended sequence shown here, the consensus sequence is embedded in the between a 5'flanking region that provides space for the T7 RNA polymerase to bind and a 3'-flanking trinucleotide that stimulates transcription catalyzed by the enzyme.

TS primer (5' AAG CAG TGG TAA CAA CGC AGA GTA CGC GGG 3') (0.25 μg/μl) SDS-PAGE purified. According to the Chenchik's [74] data, ribouncleotide GGG at the 3' end should give the best TS effect instead of deoxinucleotide GGG. We have used TS primer with dGGG at the 3' end in multiple experiments and achieved satisfying results. The amount of TS primer used in the second strand synthesis can be varied according to the amount of starting material. We generally use 0.25 μg/μl when 3–6 ug of total RNA used and 0.125 μg/μl when less total RNA used.

Random hexamer (dN_6_) (8 μg/μl).

### Columns

Micro Bio-Spin Chromatograph column (Bio-gel P-6) (Bio-Rad; Cat# 732–6222)

Microcon YM-30 column (Millipore; Cat# 42410).

#### Procedures

##### First strand cDNA synthesis

1. In PCR reaction tube, mix 0.01–5 μg total RNA in 9 μl DEPC H2O with 1 μl (0.1–0.25 μg/μl) oligo dT(15)-T7 primer (5' AAA CGA CGG CCA GTG AAT TGT AAT ACG ACT CAC TAT AGG CGC T_(15) _3'), 1 ul of RNasin Plus and heat to 70°C for 3 min. Cool to room temperature then add the following reagents: (a master mix can be prepared for multiple samples)

2. 4 μl 5 × First strand buffer

3. 1 μl (0.1–0.25 μg/μl) TS (template switch) oligo primer

4. 2 μl 0.1 M DTT

5. 2 μl 10 mM dNTP

6. 1 μl Superscript II

7. 0.5 μl of T4 gp32 (8 μg/μl)

50°C for 90 min in thermal cycler.

##### Second strand cDNA synthesis

1. Add 106 μl of DEPC treated H_2_O to the cDNA reaction tube

2. 15 μl Advantage PCR buffer

3. 3 μl 10 mM dNTP mix

4. 1 μl of RNase H

5. 3 μl Advantage cDNA Polymerase mix

Cycle at 37°C for 5 min to digest mRNA, 94°C for 2 min to denature, 65°C for 1 min. for specific priming and 75°C for 30 min for extension.

Note: Since the TS primer which initiates the second strand cDNA synthesis is already present in the first strand cDNA synthesis reaction and has been primed to the extended part of the cDNA, no additional primer is required in this step.

Stop reaction with 7.5 μl 1 M NaOH solution containing 2 mM EDTA and incubate at 65°C for 10 min. to inactivate enzymes.

(Reaction can be stopped after this step and the reaction tube can be stored at -20°C.)

##### Double stranded cDNA cleanup

(This step is designed to prevent carry over of non-incorporated dNTP, primers and inactivated enzymes into the following in *vitro *transcription. Keep in mind that although the double stranded cDNAs are stable and will not be affected by RNase contamination, they will be used as template in the IVT reaction which is RNase free.)

#### Phenol-Chloroform-Isoamyl isolation and ethanol precipitation

Add 1 μl Linear Acrylamide (0.1 μg/μl) as DNA carrier to the sample to enhance double stranded-cDNA precipitation. Add 150 μl Phenol: Chloroform: Isoamyl alcohol (25:24:1) to the double stranded cDNA tube and mix well by pipetting (be careful not to spill or contaminate). Transfer the slurry solution to Phase lock gel tube and spin at 14,000 rpm for 5 min at room temperature. Transfer the aqueous phase to RNase/DNase-free 1.7 ml tube and add 70 μl of 7.5 M ammonium acetate first and then 1 ml 100% ethanol (EtOH). Mix well. Centrifuge **right away **at 14,000 rpm for 20 min at **room temperature **to prevent co-precipitation of oligos. (A visible small white pellet should be seen at the bottom of the tube even if nano grams of starting material have been used. This pellet suggests successful precipitation.) Wash pellet with 800 μl 100% EtOH and spin down at maximum speed for 8 min. Repeat this washing step one more time. Air dry or speedvac and re-suspend double stranded cDNA in 8 ul DEPC H_2_O.

#### In Vitro Tanscription (Ambion; T7 Megascript Kit #1334)

2 μl of each 75 mM NTP (A, G, C and UTP)

2 μl reaction buffer

2 μl enzyme mix (RNase inhibitor and T7 phage RNA polymerase)

8 μl double stranded cDNA

37°C for 5 hr.

According to Ambion, the incubation can be interrupted by storing reaction tube at -20°C and resuming the incubation later without losing efficiency.

### Purification of amplified RNA

#### Any manufactured RNA isolation kit can be applied

Monophasic reagent such as TRIzol reagent from GibcoBRL, (Cat#15596) are used here based on the efficient recovery of aRNA (RNeasy mini kit could be used for aRNA purification instead of TRIzol but, in our experience, RNA recovering is about 50% of that recovered with the TRIzol method.).

a. Add 0.5 ml of TRIzol solution to the transcription reaction. Mix the reagents well by pipetting or gentle vortexing.

b. Add 100 μl chloroform. Mix the reagents by inverting the tube for 15 seconds. Allow the tube to stand at room temperature for 2 – 3 minutes.

c. Centrifuge the tube at 10,000 g for 15 min at 4°C.

d. Transfer the aqueous phase to a fresh tube and add 250 μl of isopropanol.

e. Store the sample on ice for 5 minutes and then centrifuge at 10,000 g for 15 minutes.

f. Wash the pellet twice with 800 μl 70% EtOH

g. Allow the pellet to dry in air on ice and then dissolve it in 20 μl DEPC H_2_O

h. Measure the quantity of RNA concentration spectrophotometrically.

### Second round of amplification

Mix amplified aRNA (0.5–1 μg) in 9 ul DEPC H2O with 1 μl (2 μg/μl) random hexamer (i.e. dN6) and heat to 70°C for 3 min, cool to room temperature. Then add the following reagents:

1. 4 μl 5 × First strand buffer

2. 1 μl (0.5–1 μg/ul) oligo dT-T7 primer

3. 2 μl 0.1 M DTT

4. 1 μl RNAsin

5. 2 μl 10 mM dNTP

6. 1 μl Superscript II

42°C for 90 min.

(Note: More than 1 ug of aRNA is not suggested. Too much template in IVT reaction could cause the amplification to reach a plateau with loss of amplification linearity. Because of random primer used here, 42°C in stead of 50°C is used)

From here, follow the previously described procedure for second strand cDNA synthesis, double stranded cDNA cleanup. In the second IVT, 40 ul of IVT reaction mixture are suggested to use instead of 20 ul. RNA isolation is followed.

### Target labeling by reverse transcription

4 μl First strand buffer

1 μl dN6 primer (8 μg/μl)

2 μl 10× low T – dNTP (5 mM A, C and GTP, 2 mM dTTP)

2 μl Cy-dUTP (1 mM Cy3 or Cy5)

2 μl 0.1 M DTT

1 μl RNasin

3–6 μg amplified aRNA in 8 μl DEPC H2O

Mix well and heat to 65°C for 5 min then cool down to 42°C.

Add 1.5 μl SSII. Incubate for 90 min at 42°C. Add 2.5 μl 0.5 M EDTA and heat to 65°C for 1 min. Add 5 μl 1 M NaOH and incubate at 65°C for 15 min to hydrolyze RNA. Add 12.5 μl 1 M Tris immediately to neutralize the pH. Bring volume to 70 μl by adding 35 μl of 1 × TE.

Note: The amounts of aRNA used for labeling depends on the size of the array. If the array with 2000–8000 genes, 3 ug aRNA will be sufficient while a larger chip such as 16–20 k will need 6 ug of aRNA. The labeling reaction components do not need to be changed.

#### Target clean up

Prepare Bio-6 column and run target solution through it. Collect flow through and add 250 μl 1 × TE to it. Concentrate target to ~20 μl using Microcon YM-30 column.

#### Hybridization

Combine Cy3 labeled reference sample and Cy5 labeled target sample (adjust the color to purple) and then complete dry the sample using speedvac. Resuspend sample in 37 μl volume (for 22 mm × 40 mm printing surface) containing 1 μl 50× Denhardt's blocking solution, 1 μl poly dA (8 μg/μl), 1 μl yeast tRNA (4 mg/ml), 10 μl Human Cot I DNA, 3 μl 20× SSC, 1 μl of 10% SDS and 20 μl of DEPC treated water. Heat sample for 2 min at 99°C and apply target mixture to array slide, add coverslip, place in humidified hyb chamber, and hybridize at 65°C over night.

#### Washing

1. Wash with 2 × SSC + 0.1% SDS to get rid of the cover slide.

2. Wash with 1 × SSC for 1 min.

3. Wash with 0.2 × SSC for 1 min.

4. Wash with 0.05 × SSC for 10 second

5. Centrifuge slide at 80–100 g for 3 min. (Slide can be put in slide rack on microplate carriers or in 50 ml conical tube and centrifuged in swinging-bucket rotor.)

### Scan Slide

## References

[B1] Bittner M, Meltzer P, Chen Y, Jiang E, Seftor E, Hendrix M (2000). Molecular classification of cutaneous malignant melanoma by gene expression: shifting from a countinuous spectrum to distinct biologic entities. Nature.

[B2] Wang E, Panelli MC, Marincola FM (2003). Genomic analysis of cancer. Princ Pract Oncol.

[B3] Yeoh E-J, Ross ME, Shurtleff SA, Williams WK, Patel D, Mahfouz R (2002). Classification, subtype discovery and prediction of outcome in pediatric acute lymphoblastic leukemia by gene expression profiling. Cancer Cell.

[B4] Wang E, Miller LD, Ohnmacht GA, Mocellin S, Petersen D, Zhao Y (2002). Prospective molecular profiling of subcutaneous melanoma metastases suggests classifiers of immune responsiveness. Cancer Res.

[B5] Panelli MC, Wang E, Phan G, Puhlman M, Miller L, Ohnmacht GA (2002). Genetic profiling of peripheral mononuclear cells and melanoma metastases in response to systemic interleukin-2 administration. Genome Biol.

[B6] Wang E, Lichtenfels R, Bukur J, Ngalame Y, Panelli MC, Seliger B (2004). Ontogeny and oncogenesis balance the transcriptional profile of renal cell cancer. Cancer Res.

[B7] Wang E, Ngalame Y, Panelli MC, Deavers M, Mueller P, Ju W (2005). Peritoneal and sub-peritoneal stroma may facilitate regional spread of ovarian cancer. Clin Cancer Res.

[B8] Singh D, Febbo PG, Ross K, Jackson DG, Manola J, Ladd C (2002). Gene expression correlates of clinical prostate cancer behavior. Cancer Cell.

[B9] Mellick AS, Day CJ, Weinstein SR, Griffiths LR, Morrison NA (2002). Differential gene expression in breast cancer cell lines and stroma-tumor differences in microdissected breast cancer biopsies by display array analysis. Int J Cancer.

[B10] Islam TC, Lindvall J, Wennborg A, Branden LJ, Rabbani H, Smith CI (2002). Expression profiling in transformed human B cells: influence of Btk mutations and comparison to B cell lymphomas using filter and oligonucleotide arrays. Eur J Immunol.

[B11] Sasaki H, Ide N, Fukai I, Kiriyama M, Yamakawa Y, Fujii Y (2002). Gene expression analysis of human thymoma correlates with tumor stage. Int J Cancer.

[B12] Skotheim RI, Monni O, Mousses S, Fossa SD, Kallioniemi OP, Lothe RA (2002). New insights into testicular germ cell tumorigenesis from gene expression profiling. Cancer Res.

[B13] Xiang CC, Kozhich OA, Chen M, Inman JM, Phan QN, Chen Y (2002). Amine-modified random primers to label probes for DNA microarrays. Nature Biotech.

[B14] DeRisi J, Penland L, Brown PO, Bittner M, Meltzer PS, Ray M (1996). Use of cDNA microarray to analyse gene expression patterns in human cancer. Nature Genetics.

[B15] Wang E, Marincola FM (2000). A natural history of melanoma: serial gene expression analysis. Immunol Today.

[B16] Wang E, Marincola FM (2001). cDNA microarrays and the enigma of melanoma immune responsiveness. Cancer J Sci Am.

[B17] Bonner RF, Emmert-Buck M, Cole K, Pohida T, Chuaqui R, Goldstein S (1997). Laser capture microdissection: molecular analysis of tissue. Science.

[B18] Pappalardo PA, Bonner R, Krizman DB, Emmert-Buck MR, Liotta LA (1998). Microdissection, microchip arrays, and molecular analysis of tumor cells (primary and metastases). Semin Radiat Oncol.

[B19] St Croix B, Rago C, Velculescu V, Traverso G, Romans KE, Montgomery E (2000). Genes expressed in human tumor endothelium. Science.

[B20] Tsuda H, Birrer MJ, Ito YM, Ohashi Y, Lin M, Lee C (2004). Identification of DNA copy number changes in microdissected serous ovarian cancer tissue using a cDNA microarray platform. Cancer Genet Cytogenet.

[B21] Chen JJ, Wu R, Yang PC, Huang JY, Sher YP, Han MH (1998). Profiling expression patterns and isolating differentially expressed genes by cDNA microarray system with colorimetry detection. Genomics.

[B22] Rajeevan MS, Dimulescu IM, Unger ER, Vernon SD (1999). Chemiluminescent analysis of gene expression on high-density filter arrays. J Histochem Cytochem.

[B23] Zejie Y, Xiaoyi W, Yu T, Huan H (1998). The method of micro-displacement measurement to improve the space resolution of array detector. Med Engineer Phys.

[B24] Yu J, Othman MI, Farjo R, Zareparsi S, MacNee SP, Yoshida S (2002). Evaluation and optimization of procedures for target labeling and hybridization of cDNA microarrays. Mol Vis.

[B25] Lockhart DJ, Dong H, Byrne MC, Folliette MT, Gallo MV, Chee MS (1996). Expression monitoring of hybridization to high-density oligonucleotide arrays. Nature Biotechnol.

[B26] Luo L, Salunga RC, Guo H, Bittner A, Joy KC, Galindo JE (1999). Gene expression profiles of laser-captured adjacent neuronal subtypes. Nat Med.

[B27] Trenkle T, Welsh J, Jung B, Mathieu-Daude F, McClelland M (1998). Non-stoichiometric reduced complexity probes for cDNA arrays. Nucleic Acids Res.

[B28] Van Gelder RN, von Zastrow ME, Yool A, Dement WC, Barchas JD, Eberwine JH (1990). Amplified RNA synthesized from limited quantities of heterogeneous cDNA. Proc Natl Acad Sci USA.

[B29] Eberwine JH, Yeh H, Miyashiro K, Cao Y, Nair S, Finnell R (1992). Analysis of gene expression in single live neurons. Proc Natl Acad Sci U S A.

[B30] Roozemond RC (1976). Ultramicrochemical determination of nucleic acids in individual cells using the Zeiss UMSP-I microspectrophotometer. Application to isolated rat hepatocytes of different ploidy classes. Histochem J.

[B31] Uemura E (1980). Age-related changes in neuronal RNA content in rhesus monkeys (Macaca mulatta). Brain Res Bull.

[B32] Skrypina NA, Timofeeva AV, Khaspekov GL, Savochkina LP, Beabealashvilli RS (2003). Total RNA suitable for molecular biology analysis. J Biotechnol.

[B33] Phillips J, Eberwine JH (1996). Antisense RNA amplification: a linear amplification method for analyzing the mRNA population from single living cells. Methods.

[B34] Eberwine JH (1996). Amplification of mRNA populations using aRNA generated from immobilized oligo(dT)-T7 primed cDNA. Biotechniques.

[B35] Wang E, Miller L, Ohnmacht GA, Liu E, Marincola FM (2000). High fidelity mRNA amplification for gene profiling using cDNA microarrays. Nature Biotech.

[B36] Wang E, Marincola FM, Bowtell D, Sambrook J (2002). Amplification of small quantities of mRNA for transcript analysis. DNA arrays – A Molecular Cloning Manual.

[B37] Spiess AN, Mueller N, Ivell R (2003). Amplified RNA degradation in T7-amplification methods results in biased microarray hybridizations. BMC Genomics.

[B38] Xiang CC, Chen M, Ma L, Phan QN, Inman JM, Kozhich OA (2003). A new strategy to amplify degraded RNA from small tissue samples for microarray studies. Nucleic Acids Res.

[B39] Malboeuf CM, Isaacs SJ, Tran NH, Kim B (2001). Thermal effects on reverse transcription: improvement of accuracy and processivity in cDNA synthesis. Biotechniques.

[B40] Kenzelmann M, Klaren R, Hergenhahn M, Bonrouhi M, Grone HJ, Schmid W (2004). High-accuracy amplification of nanogram total RNA amounts for gene profiling. Genomics.

[B41] Schlingemann J, Thuerigen O, Ittrich C, Toedt G, Kramer H, Hahn M (2005). Effective transcriptome amplification for expression profiling on sense-oriented oligonucleotide microarrays. Nucleic Acids Res.

[B42] Mizuno Y, Carninci P, Okazaki Y, Tateno M, Kawai J, Amanuma H (1999). Increased specificity of reverse transcription priming by trehalose and oligo-blockers allows high-efficiency window separation of mRNA display. Nucleic Acids Res.

[B43] Spiess AN, Mueller N, Ivell R (2004). Trehalose is a potent PCR enhancer: lowering of DNA melting temperature and thermal stabilization of taq polymerase by the disaccharide trehalose. Clin Chem.

[B44] Carninci P, Nishiyama Y, Westover A, Itoh M, Nagaoka S, Sasaki N (1998). Thermostabilization and thermoactivation of thermolabile enzymes by trehalose and its application for the synthesis of full length cDNA. Proc Natl Acad Sci U S A.

[B45] Rapley R (1994). Enhancing PCR amplification and sequencing using DNA-binding proteins. Mol Biotechnol.

[B46] Villalva C, Touriol C, Seurat P, Trempat P, Delsol G, Brousset P (2001). Increased yield of PCR products by addition of T4 gene 32 protein to the SMART PCR cDNA synthesis system. Biotechniques.

[B47] Baugh LR, Hill AA, Brown EL, Hunter CP (2000). Quantitative analysis of mRNA amplification by *in vitro *transcription. Nucleic Acids Res.

[B48] Gubler U, Hoffman BJ (1983). A simple and very efficient method for generating cDNA libraries. Gene.

[B49] Li Y, Li T, Liu S, Qiu M, Han Z, Jiang Z (2004). Systematic comparison of the fidelity of aRNA, mRNA and T-RNA on gene expression profiling using cDNA microarray. J Biotechnol.

[B50] Rudnicki M, Eder S, Schratzberger G, Mayer B, Meyer TW, Tonko M (2004). Reliability of t7-based mRNA linear amplification validated by gene expression analysis of human kidney cells using cDNA microarrays. Nephron Exp Nephrol.

[B51] Park JY, Kim SY, Lee JH, Song J, Noh JH, Lee SH (2004). Application of amplified RNA and evaluation of cRNA targets for spotted-oligonucleotide microarray. Biochem Biophys Res Commun.

[B52] Li Y, Ali S, Philip PA, Sarkar FH (2003). Direct comparison of microarray gene expression profiles between non-amplification and a modified cDNA amplification procedure applicable for needle biopsy tissues. Cancer Detect Prev.

[B53] Kacharmina JE, Crino PB, Eberwine JH (1999). Preparation of cDNA from single cells and subcellular regions. Methods Enzymol.

[B54] Matz M, Shagin D, Bogdanova E, Britanova O, Lukyanov S, Diatchenko L (1999). Amplification of cDNA ends based on template-switching effect and step-out PCR. Nucleic Acids Res.

[B55] Chenchik A, Zhu YY, Diatchenko L, Li R, Hill J, Siebert PD, Siebert P, Larrick J (1998). Generation and use of high-quality cDNA from small amounts of total RNA by SMART™ PCR. Gene cloning and analysis by RT-PCR.

[B56] Zhao H, Hastie T, Whitfield ML, Borresen-Dale AL, Jeffrey SS (2002). Optimization and evaluation of T7 based RNA linear amplification protocols for cDNA microarray analysis. BMC Genomics.

[B57] Marko NF, Frank B, Quackenbush J, Lee NH (2005). A robust method for the amplification of RNA in the sense orientation. BMC Genomics.

[B58] Goff LA, Bowers J, Schwalm J, Howerton K, Getts RC, Hart RP (2004). Evaluation of sense-strand mRNA amplification by comparative quantitative PCR. BMC Genomics.

[B59] Cheetham GM, Jeruzalmi D, Steitz TA (1998). Transcription regulation, initiation, and "DNA scrunching" by T7 RNA polymerase. Cold Spring Harb Symp Quant Biol.

[B60] Feldman AL, Costouros NG, Wang E, Qian M, Marincola FM, Alexander HR (2002). Advantages of mRNA amplification for microarray analysis. Biotechniques.

[B61] Gold D, Coombes K, Medhane D, Ramaswamy A, Ju Z, Strong L (2004). A comparative analysis of data generated using two different target preparation methods for hybridization to high-density oligonucleotide microarrays. BMC Genomics.

[B62] Polz MF, Cavanaugh CM (1998). Bias in template-to-product ratios in multitemplate PCR. Appl Environ Microbiol.

[B63] Seth D, Gorrell MD, McGuinness PH, Leo MA, Lieber CS, McCaughan GW (2003). SMART amplification maintains representation of relative gene expression: quantitative validation by real time PCR and application to studies of alcoholic liver disease in primates. J Biochem Biophys Methods.

[B64] Smith L, Underhill P, Pritchard C, Tymowska-Lalanne Z, Abdul-Hussein S, Hilton H (2003). Single primer amplification (SPA) of cDNA for microarray expression analysis. Nucleic Acids Res.

[B65] Bettinotti MP, Panelli MC, Ruppe E, Mocellin S, Phan GQ, White DE (2003). Clinical and immunological eveluation of patients with metastatic melanoma undergoing immunization with the HLA-C2*0702 associated epitope MAGE-A12:170–178. Int J Cancer.

[B66] Iscove NN, Barbara M, Gu M, Gibson M, Modi C, Winegarden N (2002). Representation is faithfully preserved in global cDNA amplified exponentially from sub-picogram quantities of mRNA. Nature Biotech.

[B67] Stirewalt DL, Pogosova-Agadjanyan EL, Khalid N, Hare DR, Ladne PA, Sala-Torra O (2004). Single-stranded linear amplification protocol results in reproducible and reliable microarray data from nanogram amounts of starting RNA. Genomics.

[B68] Petalidis L, Bhattacharyya S, Morris GA, Collins VP, Freeman TC, Lyons PA (2003). Global amplification of mRNA by template-switching PCR: linearity and application to microarray analysis. Nucleic Acids Res.

[B69] Rox JM, Bugert P, Muller J, Schorr A, Hanfland P, Madlener K (2004). Gene expression analysis in platelets from a single donor: evaluation of a PCR-based amplification technique. Clin Chem.

[B70] Klur S, Toy K, Williams MP, Certa U (2004). Evaluation of procedures for amplification of small-size samples for hybridization on microarrays. Genomics.

[B71] Ohtsuka S, Iwase K, Kato M, Seki N, Shimizu-Yabe A, Miyauchi O (2004). An mRNA amplification procedure with directional cDNA cloning and strand-specific cRNA synthesis for comprehensive gene expression analysis. Genomics.

[B72] Randolph JB, Waggoner AS (1997). Stability, specificity and fluorescence brightness of multiply-labeled fluorescent DNA probes. Nucleic Acids Res.

[B73] DeRisi J, Bowtell D, Sambrook J (2002). Stability, specificity and fluorescence brightness of mulitply-labeled fluorescent DNA probes. DNA arrays – A molecular cloning manual.

[B74] Chenchik A, Zhu Y Y, Diatchenko L, Li R, Hill J, Siebert PD, Siebest P. and Larrick J. , pp. (1998). Generation and use of high-quality cDNA from small amounts of total RNA by SMARTTM PCR.. Gene cloning and analysis by RT-PCR.

